# Case report: Refractory cardiac arrest supported with veno-arterial-venous extracorporeal membrane oxygenation and left-ventricular Impella CP^®^–Physiological insights and pitfalls of ECMELLA

**DOI:** 10.3389/fcvm.2022.1045601

**Published:** 2022-11-04

**Authors:** Tharusan Thevathasan, Lisa Füreder, Dirk W. Donker, Christoph Nix, Thomas H. Wurster, Wulf Knie, Georg Girke, Abdulla S. Al Harbi, Ulf Landmesser, Carsten Skurk

**Affiliations:** ^1^Department of Cardiology, Charité – Universitätsmedizin Berlin, Berlin, Germany; ^2^Berlin Institute of Health, Berlin, Germany; ^3^Deutsches Zentrum für Herz-Kreislauf-Forschung e.V., Berlin, Germany; ^4^Institute of Medical Informatics, Charité – Universitätsmedizin Berlin, Berlin, Germany; ^5^Intensive Care Center, University Medical Centre Utrecht, Utrecht, Netherlands; ^6^Cardiovascular and Respiratory Physiology, TechMed Center, University of Twente, Enschede, Netherlands; ^7^Abiomed Europe GmbH, Aachen, Germany

**Keywords:** cardiac arrest, Impella^®^, acute coronary syndrome, extracorporeal cardiopulmonary resuscitation, ECMELLA, extracorporeal membrane oxygenation, unloading

## Abstract

**Introduction:**

To the best of our knowledge, this is the first case report which provides insights into patient-specific hemodynamics during veno-arterio-venous-extracorporeal membrane oxygenation (VAV ECMO) combined with a left-ventricular (LV) Impella^®^ micro-axial pump for therapy-refractory cardiac arrest due to acute myocardial infarction, complicated by acute lung injury (ALI).

**Patient presentation:**

A 54-year-old male patient presented with ST-segment elevation acute coronary syndrome complicated by out-of-hospital cardiac arrest with ventricular fibrillation upon arrival of the emergency medical service. As cardiac arrest was refractory to advanced cardiac life support, the patient was transferred to the Cardiac Arrest Center for immediate initiation of extracorporeal cardiopulmonary resuscitation (ECPR) with peripheral VA ECMO and emergency percutaneous coronary intervention using drug eluting stents in the right coronary artery. Due to LV distension and persistent asystole after coronary revascularization, an Impella^®^ pump was inserted for LV unloading and additional hemodynamic support (i.e., “ECMELLA”). Despite successful unloading by ECMELLA, post-cardiac arrest treatment was further complicated by sudden differential hypoxemia of the upper body. This so called “Harlequin phenomenon” was explained by a new onset of ALI, necessitating escalation of VA ECMO to VAV ECMO, while maintaining Impella^®^ support. Comprehensive monitoring as derived from the Impella^®^ console allowed to illustrate patient-specific hemodynamics of cardiac unloading. Ultimately, the patient recovered and was discharged from the hospital 28 days after admission. 12 months after the index event the patient was enrolled in the *ECPR Outpatient Care Program* which revealed good recovery of neurologic functions while physical exercise capacities were impaired.

**Conclusion:**

A combined mechanical circulatory support strategy may successfully be deployed in complex cases of severe cardio-circulatory and respiratory failure as occasionally encountered in clinical practice. While appreciating potential clinical benefits, it seems of utmost importance to closely monitor the physiological effects and related complications of such a multimodal approach to reach the most favorable outcome as illustrated in this case.

## Introduction

Extracorporeal cardiopulmonary resuscitation (ECPR) is the initiation of veno-arterial extracorporeal membrane oxygenation (VA ECMO) during refractory cardiac arrest, that is failure to obtain return of spontaneous circulation (ROSC) after prolonged conventional resuscitation attempts (1, 2). The use of VA ECMO enables clinicians to establish a “bridge to therapy” (such as emergency coronary angiography/intervention), “bridge to decision” or “bridge to recovery” while enabling proper organ perfusion. ECPR has been recently shown to have the potential to improve survival and/or neurological outcome compared to conventional CPR (3, 4). Given its relative ease of implantation and immediate provision of full circulatory support, ECPR with VA ECMO has gained wider application in carefully selected patients, with a 10-fold increase in use between 2003 and 2014. ECPR has been advocated in recent international guidelines (5–8). Very recently, adjunct left-ventricular (LV) unloading with an Impella^®^ micro-axial flow pump (Abiomed, Danvers, USA) as an adjunct to VA ECMO (so called “ECMELLA” or “ECPELLA”) has been proposed during ECPR. ECMELLA has been shown to be associated with improved survival after therapy-refractory cardiac arrest, possibly by mitigating inherent shortcomings of VA ECMO, such as hemodynamic overload of the left ventricle (9–11).

To our best knowledge, this is the first case report to comprehensively illustrate critical insights into hemodynamic monitoring as readily accessible after ECPR and immediate LV unloading by Impella^®^. The integration of all vital monitoring information sets the stage for optimal, patient-specific tailoring of mechanical circulatory support (MCS) at the bedside throughout the course of critical care management. This is also reflected by a successful escalation of MCS to Impella^®^ and veno-arterial-venous ECMO (VAV ECMO) to control the complication of sudden differential hypoxemia of the upper body due to acute lung injury (ALI). As “physiology on display” this case illustrates the complex hemodynamic principles of combined MCS and informs the clinician on the adequacy of the conceived clinical management at the bedside. Close monitoring allows for timely detection of suboptimal MCS, related complications and eventually the initiation of additional interventions, all being critical elements for a successful deployment of ECPR.

## Case description

A 54-year-old male patient presented with acute onset of chest pain, severe dyspnea, sweating, anxiety and arterial hypotension at his home. ST-segment elevations in leads II, III, and aVF were detected on the first electrocardiogram (ECG) by the emergency medical service (EMS). Apart from smoking (equivalent to 24 pack years), the patient’s history did not reveal additional cardiovascular risk factors and his family history was unremarkable.

**FIGURE 1:**
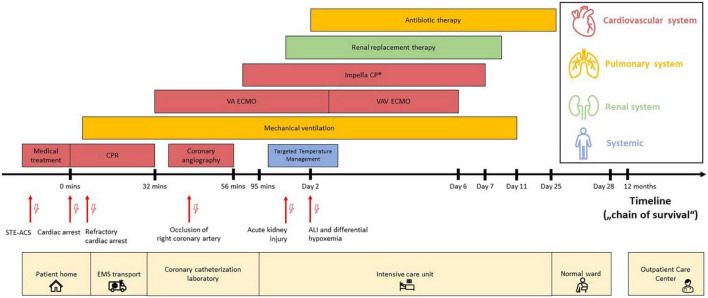
Timeline (“chain of survival”) with data on the episodes of care. The timeline (“chain of survival”) reflects the pre-, intra- and post-hospital course of the cardiac arrest patient, which includes the period from onset of patient symptoms for suspected STE-ACS to hospital discharge and follow-up assessment in the *ECPR Outpatient Care Program* of the Cardiac Arrest Center. Each location is displayed at the bottom (light yellow color). Each critical clinical diagnosis is highlighted with red arrows (and lightning symbols) at the timeline. The provided medical procedures are colorized by organ system: cardiovascular system (red), pulmonary system (dark yellow), renal system (green) and non-specific (blue). The image scaling does not correlate with the actual time. Abbreviations: acute lung injury (ALI); cardiopulmonary resuscitation (CPR); emergency medical service (EMS); left ventricle (LV); ST-segment elevation acute coronary syndrome (STE-ACS); veno-arterial extracorporeal membrane oxygenation (VA ECMO); veno-arterio-venous ECMO (VAV ECMO).

**FIGURE 2:**
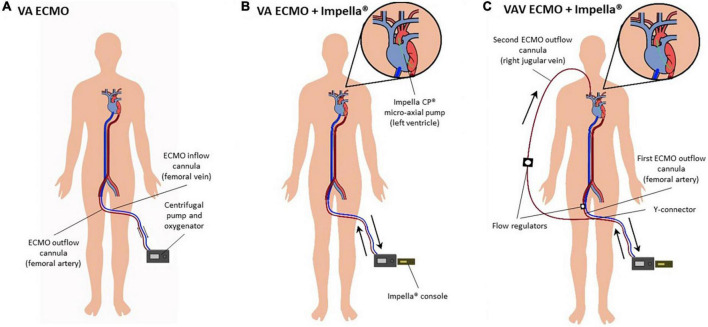
**(A–C)** Diagram on configurations of percutaneous mechanical circulatory support devices. **(A)** Peripheral VA ECMO. Poorly oxygenated blood is drawn from the femoral vein, oxygenated in a membrane oxygenator and returned by a centrifugal pump via the common femoral artery. **(B)** VA ECMO and Impella^®^ pump (“ECMELLA”). The Impella^®^ pump is placed across the aortic valve to provide continuous blood flow from the LV into the proximal ascending aorta. **(C)** VAV ECMO and Impella^®^. VAV ECMO is a triple cannulation technique by “upgrading” the VA ECMO configuration. A third cannula is inserted into the jugular or subclavian vein. The ECMO outflow with oxygenated blood is diverted into two cannulas by a Y-connector, one cannula toward the aorta via the common femoral artery and one toward the right atrium via the jugular or subclavian vein. Flow regulators control blood flow into the arterial and venous circuit, respectively. Abbreviations: left ventricle (LV); veno-arterial extracorporeal membrane oxygenation (VA ECMO); veno-arterio-venous ECMO (VAV ECMO).

In the presence of the EMS, the patient collapsed due to ventricular fibrillation. Immediate CPR was commenced on scene (i.e., no no-flow time) in accordance with current guidelines on advanced cardiac life support (ACLS). Mechanical ventilation was provided with synchronized intermittent mechanical ventilation (SIMV). After 15 min of refractory CPR, the EMS decided to transport the patient to the Cardiac Arrest Center by utilizing a mechanical resuscitation device (CorPulse^®^, GS Elektromedizinische Geräte G. Stemple GmbH) as a potential candidate for ECPR since all ECPR criteria were fulfilled (7, 12). Upon arrival in the cardiac catheterization laboratory, the patient’s initial pH was 6.67, serum lactate level 88 mg/dL, partial pressure of CO_2_ 71 mmHg and end-tidal CO_2_ 13 mmHg. According to current guidelines, 10 electrical defibrillations, 7 mg epinephrine and 450 mg amiodarone were administered in total. Upon discretion of the ECPR team, VA ECMO support was initiated after 32 min of total low-flow time (Figures 1, 2A). The cardiac rhythm deteriorated from persistent ventricular fibrillation to asystole upon VA ECMO cannulation.

**FIGURE 3:**
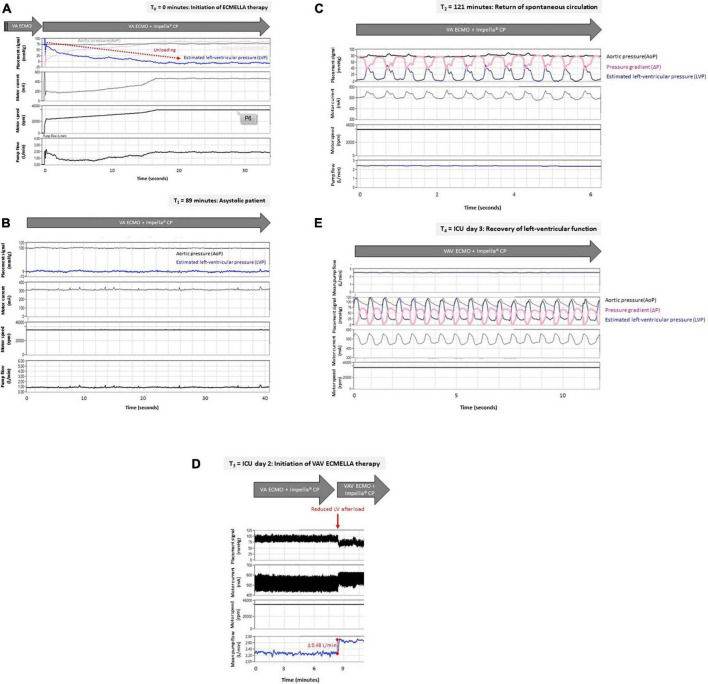
**(A–E)** Left-ventricular unloading in a patient with out-of-hospital cardiac arrest treated with VAV ECMO and Impella^®^ pump. Raw data were retrospectively retrieved from the Impella CP^®^ console (Abiomed, Danvers, USA). For each given time point after initiation of ECMELLA therapy (T0) the estimated left-ventricular pressure (LVP in mmHg), aortic pressure (AoP in mmHg, derived from Impella^®^ pressure sensor) or mean arterial pressure (MAP in mmHg), as well as Impella^®^ motor current (milliampere, mA), Impella^®^ motor speed (rotations per minute, rpm) and Impella^®^ pump flow (liters per minute, L/min) are displayed. LVP was estimated based on AoP and pressure gradient (ΔP in mmHg, derived from Impella^®^ motor current) as follows: LVP = AoP −ΔP. **(A)** Impella CP^®^ was inserted transfemorally in an asystolic patient with pre-existing VA ECMO. Displayed are the first 34 s after start of Impella CP^®^ indicated by increasing motor current and motor speed. Depending on the Impella CP^®^ flow (up to 2 L/min), the estimated LVP decreased to 9 mmHg (i.e., LV unloading indicated by red arrow). Therefore, despite asystole, the patient exhibited physiologic AoP (i.e., 75 mmHg) provided by VA ECMO flow of 4 L/min with sufficient unloading of the LV (physiologic LVP – LVEDP of 9 mmHg). **(B)** The patient remained in asystole without ROSC for approximately two hours after initiation of ECMELLA therapy indicated by static AoP and estimated LVP (no pulsatility). In this case, an Impella^®^ pump flow of 1 L/min was sufficient to unload the LV (estimated LVP, i.e., LVEDP under asystole of 9mmHg) while a VA ECMO flow of 4 L/min provided a sufficient hemodynamic support (i.e., MAP 100 mmHg). **(C)** ROSC with sinus rhythm was achieved after approximately two hours following initiation of ECMELLA therapy as being indicated by undulating estimated LVP (pulsatility indicating cardiac contractions). Flat AoP (greater than estimated LVP) indicates lack of aortic valve opening. Of note, Impella^®^ pump flow was set to 2.5 L/min which reduced LV preload. VA ECMO flow was set to 4 L/min. **(D)** Two days after ICU admission, VA ECMO therapy was advanced to VAV ECMO therapy while the Impella^®^ pump remained inserted. Induction of VAV ECMO therapy (i.e., reduction of ECMO outflow to the aorta and therefore reduction in LV afterload) was associated with increased mean Impella^®^ pump flow (increase in Impella^®^ pump flow of 0,48 L/min, indicated by red line) with given constant pump settings (Impella^®^ motor current). Impella pump flow was 2.7 L/min, VA ECMO flow was 2.5 L/min and VAV ECMO flow was 1.5 L/min. **(E)** Differential hypoxemia was successfully reversed by VAV-ECMELLA. LV function fully recovered over the next days. Estimated LVP was now greater than AoP resulting in opening of aortic valve and pulsatile aortic pressures (MAP 75 mmHg) despite unloading (Impella^®^ pump flow 2 L/min). ECMO flow was 1.5 L/min. Abbreviations: ECMELLA (VA ECMO plus Impella CP^®^); intensive care unit (ICU); left-ventricular (LV); return of spontaneous circulation (ROSC); veno-arterial extracorporeal membrane oxygenation (VA ECMO); VAV-ECMELLA (VAV ECMO plus Impella CP^®^).

Emergent coronary angiography under VA ECMO revealed a proximal occlusion of the right coronary artery, which was successfully revascularized with two drug eluting stents. Echocardiographic LV distension was detected in the asystolic patient, wherefore a LV micro-axial pump (Impella CP^®^) was inserted transfemorally to ensure LV unloading and increase cardiac output (Figures 1, 2B) (ECMELLA). Upon addition of Impella^®^ support, the estimated mean intracavitary left-ventricular pressure (LVP) decreased from 78 to 9 mmHg within 20 seconds (Figure 3A). Despite coronary revascularization, the patient remained in asystole while ECMELLA provided adequate systemic hemodynamic support, i.e., aortic pressure of approximately 70mmHg, while assuring optimal LV loading conditions at a level comparable to physiological diastolic LVPs (Figure 3B).

The patient was then admitted to the cardiac intensive care unit (ICU) for post-resuscitation care (13) including targeted temperature management, circulatory and respiratory management (e.g., norepinephrine infusion), as well as renal replacement therapy due to acute kidney injury. ROSC (sinus rhythm with pulsatile LVPs) was ultimately observed approximately two hours after ECMELLA initiation (Figure 3C).

**FIGURE 4:**
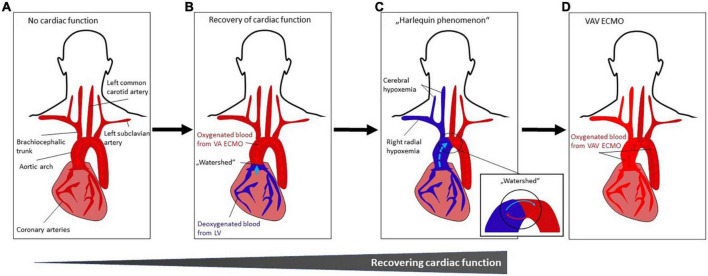
**(A–D)** Occurrence and treatment of differential hypoxemia during VA ECMO treatment**. (A)** *No cardiac function.* Systemic perfusion with oxygenated blood (indicated by dark red color) is solely dependent from VA ECMO flow which is infused retrogradely toward the heart. **(B)** Recovery of cardiac function with impaired pulmonary gas exchange. The LV starts ejecting poorly oxygenated blood from the pulmonary circulation (indicated by blue color) that mixes with oxygenated blood from the VA ECMO (“watershed” or “mixing cloud”). The location of the “watershed” depends on the LV function and the VA ECMO flow. With severe myocardial dysfunction (e.g., due to cardiac arrest), the watershed is close to or at (if asystolic patient) the aortic valve. **(C)** *Harlequin phenomenon.* With improving LV function, the “watershed” may move more distally in the aortic arch. As a result, given impaired pulmonary function, poorly oxygenated blood from the LV is ejected into the aortic arch, coronary and cerebral arteries, resulting in ischemia and cyanosis of the upper body (in this case, right upper body), while the lower body is sufficiently oxygenated by the VA ECMO circuit. This phenomenon is known as *differential hypoxemia*, *watershed phenomenon*, *two-circulation syndrome* or *Harlequin phenomenon*. **(D)** *VAV ECMO.* The Harlequin phenomenon (Figure 3C) can be treated by converting VA ECMO to VAV ECMO. Thus, ECMO-oxygenated blood (indicated by lighter red color) is infused from a third ECMO cannula via the jugular or subclavian vein through and the pulmonary circulation into the LV and upper body (see Figure 1C). Abbreviations: left ventricle (LV); veno-arterial extracorporeal membrane oxygenation (VA ECMO); veno-arterio-venous ECMO (VAV ECMO).

On ICU day two, near-infrared spectroscopy (NIRS) indicated a decline in cerebral oxygen saturation during appropriate LV unloading. A chest X-ray showed bilateral pulmonary infiltrates compatible with ALI, most likely caused by a combination of aspiration pneumonia, severe transient pulmonary congestion and ischemia-reperfusion-related pulmonary inflammation. Standardized management was initiated in accordance with current ALI/ARDS and ECPR guidelines, including broad spectrum antibiotics and lung-protective mechanical ventilation (pressure-controlled ventilation mode) during ECMO treatment with low tidal volumes (6-8 ml/kg ideal body weight) and minute ventilation (i.e., avoidance of barotrauma) and with high positive end-expiratory pressures (PEEP) of at least 12 mmHg (i.e., maintenance of alveolar inflation) (7, 14). Prone positioning was not performed due to combined MCS. NIRS readings and right radial arterial blood oxygen saturation remained impaired, indicating hypoxemia of the upper body compatible with differential hypoxemia (Figures 4A–C). Optimization of ventilator settings were not sufficient, wherefore – according to guidelines by the Extracorporeal Life Support Organization (ELSO) guidelines – VA ECMO was converted to VAV ECMO via the right jugular vein, while continuing concomitant Impella^®^ support (Figure 2C) (7).

As a result, NIRS readings and right radial oxygen saturations normalized (Figure 4D). The initiation of VAV ECMO resulted in a reduction of extracorporeal support flow into the arterial part of the systemic circulation, as the total flow of arterialized extracorporeal blood was divided into a venous and arterial limb of the VAV ECMO circuit. This resulted in reduced LV afterload and increased Impella^®^ pump flow (Figure 3D). Finally, the patient recovered clinically and hemodynamically including increasing LVPs, aortic valve re-opening and improved LV ejection fraction (LVEF) (Figure 3E), allowing for stepwise weaning from VAV ECMO and Impella^®^.

Metabolic homeostasis, i.e., pH and lactate levels, was reached within days, while kidney function recovered and infection, i.e., leukocytes and C-reactive protein levels, diminished after antibiotic treatment (Supplementary Figure 1 in Supplementary material). The patient was discharged from the hospital with a good neurologic outcome (classified as a Cerebral Performance Category [CPC] scale of 1) on day 28. Follow-up in the *ECPR Outpatient Care Program* of the Cardiac Arrest Center after 12 months following the index event showed good neurological recovery but impaired exercise capacities (see “Patient Perspective”) (15).

## Discussion

Although ECPR has recently gained wider attention with increasing application rates, patient selection, duration of cannulation, as well as survival and neurologic outcomes vary widely across institutions (1). Careful patient selection is crucial for successful outcomes but remains challenging in emergency settings with remarkable time constraints and lack of information on patient history. Based on international guidelines, ECPR should be considered in carefully selected patients based on generally available parameters, such as patient age ≤70 years, witnessed cardiac arrest, low-flow time ≤60 min, no-flow time ≤5 min and exclusion of asystole on the initial ECG, active bleeding or terminal illness (7, 12).

In the presence of specific EMS infrastructures and adequate adherence to aforementioned selection criteria, ECPR has great potential to significantly improve patient survival in dedicated centers. However, MCS remains a complex and demanding technique requiring solid logistics and multidisciplinary expertise. To provide a successful ECPR program including critical care management, it is of pivotal importance to gather and maintain profound knowledge on the physiology and peculiarities of different MCS modalities including combined support strategies among healthcare providers involved in patients’ daily management. This case comprehensively illustrates the spectrum of critical hemodynamic and related critical care challenges of VA ECMO and VAV ECMO support and adjunct use of Impella^®^.

### Veno-arterial extracorporeal membrane oxygenation

In this clinical case, cardiac arrest was refractory to conventional resuscitation attempts requiring ECPR with VA ECMO prior to coronary revascularization.

#### Technique

There are two VA ECMO cannulation principles - peripheral and central (16).

##### Peripheral cannulation

Peripheral VA ECMO is the preferred strategy for ECPR due to its relative ease and rapidity of cannulation. Through a venous cannula (femoral vein, multistage cannula in superior/inferior vena cava and right atrium), blood is drained from the venous system by an extracorporeal centrifugal pump, oxygenated in a membrane oxygenator and returned to the arterial circulation via a cannula in the common femoral artery (Figure 2A). Notably, VA ECMO blood flow into the femoral artery and aorta is continuous and directed retrogradely as compared to physiological conditions, both potentially contributing to an increased cardiac afterload. VA ECMO allows for systemic circulation with oxygenated blood to adequately perfuse all end-organs after cardiac arrest, commonly striving for 4 L/min in adults. Due to the lack of palpable femoral pulsatility during cardiac arrest, ultrasound- or fluoroscopic-guided cannulation or surgical cut-down should be considered to facilitate cannulation, which is well possible in peripheral VA ECMO under conditions of on-going CPR.

##### Central cannulation

In central VA ECMO, at least one of the cannulas (venous or arterial) is placed directly in a cardiac chamber or central vessel (i.e., vena cava, pulmonary artery or aorta). Central VA ECMO necessitates open cardiac surgery and allows to obtain higher VA ECMO flows due to shorter and larger bore cannulas and is generally considered in the context of cardiac surgery or if peripheral cannulation is deemed impossible.

#### Relevant physiological considerations

Given the continuous and retrograde nature of aortic perfusion in peripheral VA ECMO, an increased LV afterload and related stresses on the myocardium may commonly occur. Hence, the LV pressure-volume loop is shifted upwards and rightwards (Supplementary Figure 2) (17). In more detail, higher LV end-diastolic volumes (LVEDV) and pressures (LVEDP) can be observed, as being described in our clinical case (Figure 3A). This may formally be translated into increased stroke work and enhanced myocardial oxygen demand, especially when considering that increased myocardial stresses may also compromise myocardial perfusion and further reduce myocardial oxygen delivery. LV hemodynamic overload might result in increased left atrial and pulmonary capillary wedge pressures (PCWP) that, in turn, promote unwanted pulmonary congestion and impaired pulmonary oxygen exchange. This creates a vicious circle, where LV contractility may potentially further be impaired cumulating into a progressive inability to eject blood against an increased LV afterload. In case of severe LV dysfunction, as commonly encountered following cardiac arrest, a condition of severe hemodynamic LV overload may occur. Without urgent correction, this may deteriorate toward life-threatening conditions such as aortic valve standstill, impending LV cavity thrombosis. Furthermore, hydrostatic pulmonary edema as well as ALI may ensue, the latter further complicating adequate pulmonary gas exchange and tissue perfusion, which in combination with ventricular arrhythmias and overload-induced irreversible myocardial damage or hampered recovery may jeopardize a good clinical outcome (1).

On the other hand, if cardiac function starts recovering while the pulmonary gas exchange remains impaired due to a complex multi-mechanistic ALI, the LV will potentially eject poorly oxygenated blood into the aorta that will mix with the properly oxygenated ECMO-derived blood. The retrogradely infused blood arising from the VA ECMO circuit and entering the aorta toward the heart meets the blood that is (poorly) oxygenated in the lungs and ejected by the native heart (“watershed” or “mixing cloud”) (Figures 4A–C). The location of the “watershed” depends on the delicate balance between the native LV function and the degree of VA ECMO flow. With severe myocardial dysfunction (as being observed after cardiac arrest), the watershed is close to or at the aortic valve (e.g., in an asystolic patient). With improving LV function the watershed may move more distally within the aortic arch. If patients on VA ECMO develop severe pneumonia, pulmonary edema or ALI, oxygenation of blood in the pulmonary circulation is often severely impaired. As a result, poorly oxygenated blood is ejected from the LV into the aortic arch, coronary and cerebral arteries, resulting in hypoxemia of the upper body, while the lower body is sufficiently oxygenated through the VA ECMO circuit. This phenomenon is known as *differential hypoxemia*, *watershed phenomenon*, *two-circulation syndrome* or *Harlequin syndrome* and has important clinical implications. Conversion of VA ECMO to VAV ECMO needs to be considered in this setting (Figure 4D).

#### Clinical considerations

Signs of LV overload should be monitored based on serial non-invasive and invasive methods, including clinical examination (increased ventilation efforts, tracheal secretions, bloody or watery sputum), vital signs (hypoxemia), transthoracic echocardiography (increased LV end-diastolic dimensions, decreased LVEF, mitral valve regurgitation or impaired aortic valve opening), chest radiography (pulmonary congestion) and pulmonary artery catheter measurements (pulmonary artery pressure, increased PCWP and mean right atrial pressure). Recovery of cardiac function is indicated by increasing pulse pressure and MAP, whereas deterioration of cardiac function is indicated by the opposite combined with increasing LVEDP and PCWP (18).

Of note, VA ECMO support requires systemic anticoagulation wherefore clinicians should closely monitor signs of bleeding. On the other hand, the large cannulas may increase the risk of limb ischemia and venous thrombosis. VA ECMO is (relatively) contraindicated in patients with aortic valve insufficiency, aortic dissection and high bleeding risk. Perfusion of the extremities and the brain should be carefully monitored (for example by NIRS) to detect differential hypoxemia. An antegrade cannula should be inserted distally to the insertion point of the arterial ECMO cannula to provide antegrade limb perfusion.

### Left-ventricular unloading with Impella^®^

In this clinical case, potent LV unloading during VA ECMO was successfully performed by concomitant use of Impella CP^®^.

#### Technique

In patients with LV overload, timely mechanical LV unloading should be considered. There are multiple strategies for percutaneous or surgical unloading (2, 19). LV unloading with an Impella^®^ micro-axial flow pump is considered to be one of the most potent and widely accepted strategies when deploying percutaneous MCS devices. The Impella^®^ pump is placed across the aortic valve to provide continuous blood flow from the LV into the proximal ascending aorta and, thus, allows to decrease LV volume and pressure (Figure 2B). The correct positioning of the Impella^®^ pump can be easily confirmed with bedside echocardiography or fluoroscopy. Different types of Impella^®^ pumps are available, providing different levels of hemodynamic support: Impella 2.5 (maximum flow rate 2.5 L/min); Impella CP (3.0-4.0 L/min); Impella 5.0 (5.0 L/min; surgical insertion) and Impella 5.5 (up to 6.0 L/min; surgical insertion).

#### Relevant physiological considerations

The direct hemodynamic effects of ECMELLA are the loss of isovolumetric periods due to continuous Impella^®^ blood flow from the LV into the aorta throughout the cardiac cycle. Hence, the pressure-volume loop is shifted to the left (LV unloading) and its shape is changed from trapezoid to triangular (Supplementary Figure 2) (17). In principle, the Impella^®^ pump causes three main effects:

##### Increase in cardiac power output

Given the pump’s support level (P level) and the pressure gradient between the aorta and LV (generated against an increased LV afterload in the setting of VA ECMO support), the Impella^®^ provides an active increase in forward flow, as demonstrated in this case (Figure 3A).

##### Increase in oxygen supply

The blood flow in the coronary arteries is determined by the pressure gradient across the coronary arterial system and its related vascular resistance. Assuming that the venous pressure and the resistance of the primary arterial vascular bed is fixed under ischemic conditions of an altered autoregulation, the coronary artery flow will largely depend on the aortic pressure. While Impella^®^ augments pressure in the ascending aorta, it promotes LV unloading by reducing LVEDP and LVEDV, which also reduces stresses imposed on the myocardium, generally dictated by Laplace’s Law. Therefore, hemodynamic support with Impella^®^ has the potential to favorably alter the myocardial oxygen supply. Moreover, it should be noted that a reduction of LVEDP and PCWP will likely contribute to an improved pulmonary oxygen exchange, breaking the vicious circle.

##### Decrease in oxygen demand

The Impella^®^ reduces LVEDP and LVEDV leading to reduced myocardial mechanical loading conditions. The reduction of preload will reduce contractility based on the Frank-Starling mechanism, leading to reduced mechanical work and lowered myocardial oxygen demand. Taken together, the delicate balance between myocardial oxygen demand and supply is likely to be favorably altered in an individual patient when deploying Impella^®^ as an adjunct to VA ECMO and when comprehensive bedside monitoring allows individualized tailoring of MCS as based on mechanistic insights.

#### Clinical considerations

While the use of ECMELLA has been extensively shown to be a practically very feasible and effective strategy in cardiogenic shock in humans and in animal models (20–23) research in the field of ECPR is urgently required. There are presently no established recommendations for LV unloading during therapy-refractory cardiac arrest, although multiple centers have been utilizing this strategy in the setting of ECPR (9–11). Currently, the decision for Impella^®^ unloading is left to the discretion and experience of the ECPR team based on clinical, hemodynamic, radiographic and echocardiographic parameters.

Limited hemodynamic support, contraindications (such as aortic stenosis, prosthetic aortic valve, LV thrombus or ventricular septal defect) and higher treatment costs should be considered when performing LV unloading with Impella^®^. Clinicians should also carefully monitor signs and markers of hemolysis which might be due to increased levels of shear stress in the Impella^®^ micro-axial pump. An antegrade perfusion cannula at the Impella^®^ insertion site is rarely needed due to the small size of the Impella^®^ access.

### Veno-arterial-venous extracorporeal membrane oxygenation

In this clinical case, the patient developed ALI while cardiac function was recovering. Deterioration of pulmonary gas exchange resulted in diminished NIRS readings and right radial hypoxemia. Therefore, treatment was converted from VA ECMO to VAV ECMO.

#### Technique

VAV ECMO is a triple cannulation technique utilized for patients who either develop differential hypoxemia (e.g., due to severe lung failure) on VA ECMO for heart failure support or, vice versa, who develop heart failure on VV ECMO for lung failure support. In this case, VAV ECMO was an “upgrade” from VA ECMO by insertion of a third cannula into the right jugular vein (Figure 2C). The venous drainage cannula in the femoral vein drains blood from the inferior caval vein, while the ECMO outflow with oxygenated blood is diverted into two cannulas by a Y-connector, with one leading toward the aorta through the femoral artery and one toward the right atrium through the jugular or subclavian vein. Consequently, VAV ECMO additionally provides oxygenated blood (from the arterial limb of the ECMO circuit) to the pulmonary, coronary and cerebral circulations.

#### Relevant physiological considerations

Since ECMO outflow is diverted to the aorta and the jugular vein, the total amount of retrograde aortic flow to the heart is reduced accordingly, wherefore LV afterload tends to be reduced. As a consequence, LVP decreased and Impella^®^ pump flow increased after conversion to VAV ECMO therapy (Figure 3D).

#### Clinical considerations

In this clinical case, VAV ECMO aided to counteract differential hypoxemia in the patient’s upper body since ECMO-oxygenated blood was ejected from the LV (Figure 4D). The diversion of the ECMO outflow cannulas has to be monitored with flow sensors on the circuit limbs and balanced with adjustable clamps since important changes in flow balance may affect oxygen saturation, preload, afterload and the position of the watershed. Serial echocardiographic monitoring as well as oxygenation monitoring of the upper (NIRS and right radial artery) and lower body (in case of impaired function of the membrane oxygenator) are crucial to assess right and left ventricular function, as well as adequate tissue oxygenation.

### Current trends in cardiopulmonary resuscitation

Apart from ECPR, recent international guidelines propose a centralized care of cardiac arrest patients in specialized Cardiac Arrest Centers (24). Hence, ECPR programs have been designed in metropolitan areas to facilitate ECPR in selected patients, such as the “Minnesota Mobile Resuscitation Consortium” (25). Importantly, in order to further improve the chain of survival, recent international guidelines propose the raise of CPR awareness and involvement of communities, such as community responders and telephone-guided CPR (24).

The time period between cardiac arrest and onset of ECPR is a crucial determinant for patient survival (26, 27). The ideal therapeutic window for ECPR has been propagated to be within 60 minutes after patient collapse (28). Low-flow times in out-of-hospital cardiac arrest (OHCA) are generally longer than in in-hospital cardiac arrest (IHCA) due to the time required to provide conventional advanced life support on scene, transport time to the ECPR center and duration of VA ECMO implantation (3, 4, 29, 30). In order to shorten low-flow times, multiple pre-hospital ECPR programs have been established to increase ECMO accessibility for patients with OHCA distant to ECPR centers. For example, a vehicle-based and helicopter-borne ECPR program were introduced in the Paris area with an average low-flow time of 57 minutes and 110 minutes, respectively (31, 32). Additionally, multiple case reports and series on pre-hospital ECPR have been published (33–35) in addition to several forthcoming (pre-hospital) ECPR trials (36–38).

Of note, low-flow time in this case was considerably low due to geographic proximity to the Cardiac Arrest Center, pre-hospital alert of the ECPR team with timely protocol-based preparations (including immediate ECMO priming), in-hospital availability of a senior interventional cardiologist with extensive ECPR case volumes and an uncomplicated percutaneous femoral access with ultrasound and fluoroscopic guidance in a rather young, non-obese patient.

Despite the current trends, data on ECPR selection criteria are still lacking. Selection criteria for ECPR with highest survival probability have been proposed by international societies (7, 12), such as witnessed cardiac arrest, no-flow time of less than 5 min, low-flow time of less than 60 min, high-quality CPR and reversible underlying cause of cardiac arrest (such as ACS). ECPR is not recommended for example when pH is <6.8 or lactate >180 mg/dL, as well as when patients are older than 70 years or have life-limiting comorbidities. The low pH level in this case presentation (pH 6.67) was in part due to hypercapnia (pCO_2_ 71 mmHg) with a lactate of 88 mg/dL in the first blood gas analysis indicating temporary hypoventilation rather than very low flow, while all other ECPR criteria were fulfilled wherefore ECPR was considered to be suitable for this young patient.

### Patient’s perspective

The patient was followed-up in the *ECPR Outpatient Care Program* after 12 months (Figure 1). He showed good recovery of neurological function while physical exercise capacity was still limited. Moreover, the patient reported depressive symptoms and mild impairment of memorizing new information. He required help to plan his daily life and has not yet been able to return to work as a machinist. Yet, significant impairments in performance of activities of daily living (Barthel Index: 100/100 points; modified Rankin Scale: 1/6 points) and cognitive function (Mini–Mental State Examination: 26/30 points; CPC scale: 1/5 points) could not objectively be quantified using generally accepted measuring tools.

Additionally, the patient reported dyspnea on exertion. While the vital parameters were unremarkable at rest, the bicycle ergometry was terminated prematurely after approximately 3.5 minutes at 75 Watts (38% of target value). The “six-minute walk test” and the “timed up and go test” showed impaired results with 229 meters of total walking distance and 18 s of total time period, respectively. The LVEF was preserved with 56% and echocardiographic myocardial strain analysis was unremarkable.

## Conclusion

This clinical case exemplifies that alongside relevant clinical and physiological bedside observations, a patient-specific, combined MCS strategy may successfully be deployed in complex scenarios of severe cardio-circulatory and respiratory failure that may arise during and after cardiac arrest. It is of utmost importance to closely integrate all clinical monitoring information that is gathered in the catheterization laboratory and the critical care unit to allow for timely detection of physiological pitfalls and related clinical complications of such multimodal approach to achieve the best patient outcomes after ECPR.

## Data availability statement

The original contributions presented in this study are included in the article/Supplementary material, further inquiries can be directed to the corresponding author/s.

## Ethics statement

Written informed consent was obtained from the patient for the publication of any potentially identifiable images or data included in this article.

## Author contributions

TT, LF, and CS: conception and design. TT, LF, DD, CN, TW, WK, GG, AA, and CS: data collection and interpretation of data. TT, LF, DD, TW, WK, GG, AA, UL, and CS: drafting of the manuscript or revising it critically for important intellectual content. TT, LF, DD, CN, TW, GG, AA, UL, and CS: final approval of the manuscript submitted.
