# Supplementary material for: Case report: Refractory cardiac arrest supported with veno-arterial-venous extracorporeal membrane oxygenation and left-ventricular Impella CP®–Physiological insights and pitfalls of ECMELLA
Source: Front Cardiovasc Med. 2022 Nov 4;9:1045601. doi: 10.3389/fcvm.2022.1045601 (PMC9674118; doi:10.3389/fcvm.2022.1045601)
Supplement: Supplementary file 1 [file Data_Sheet_1.pdf]

Supplementary Material

Supplementary Figure 1. Laboratory values during hospital stay of the patient

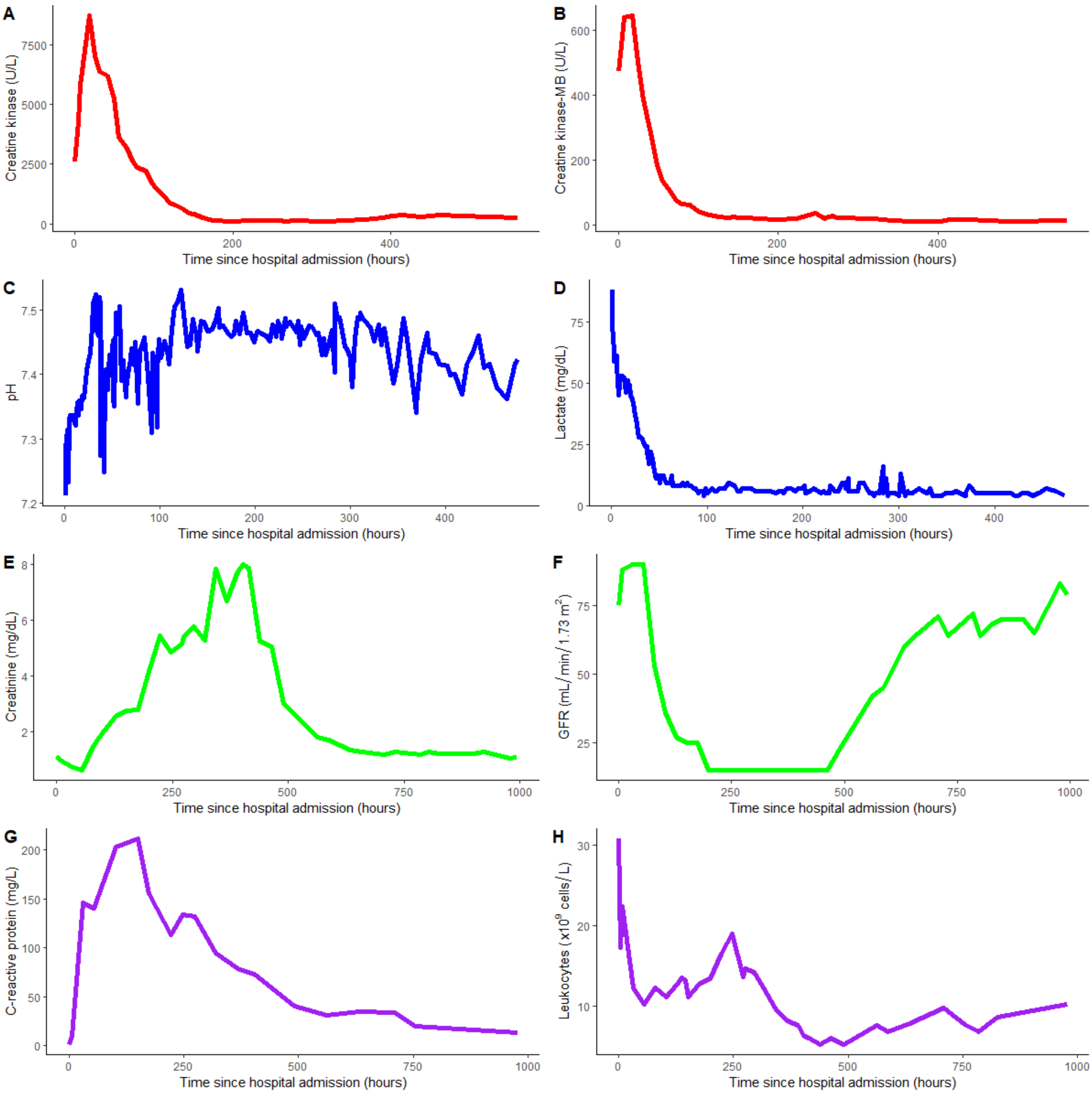

Abbreviations: glomerular filtration rate (GFR)

Supplementary Figure 2. Pressure-volume loops of the left ventricle during (concomitant) treatment with percutaneous mechanical circulatory support devices

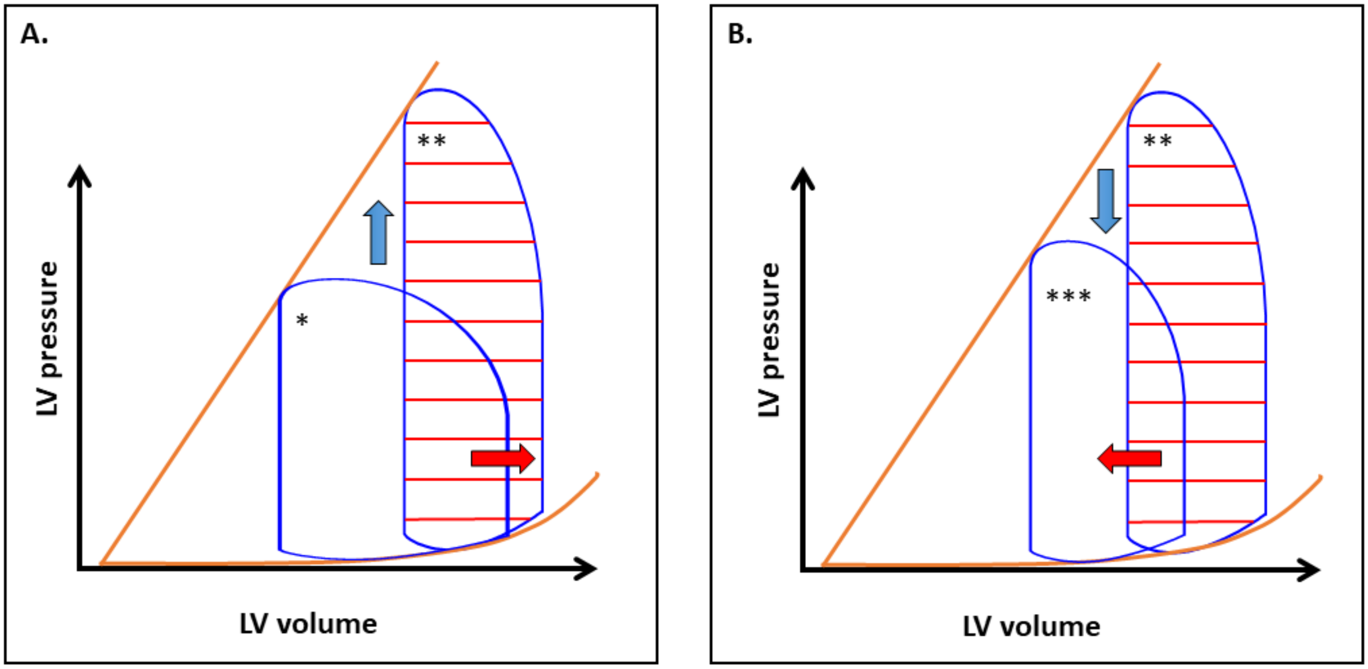

A. Pressure–volume loops during normal cardiac activity as reference (*) and after initiation of VA-ECMO support (**). The blue arrow indicates an increase in wall stress and afterload by VA-ECMO; the shift of the pressure-volume loop to the right (red arrow) indicates increased energy demand.

B. Pressure–volume loop after insertion of Impella® micro-axial pump concomitant to VA-ECMO (***). The red arrow indicates left-ventricular unloading (i.e., reduced energy demand). The blue arrow indicates reduced afterload.

Abbreviations: left ventricle (LV); veno-arterial extracorporeal membrane oxygenation (VA-ECMO).
